# Effects of glucose ingestion at different frequencies on glycogen recovery in mice during the early hours post exercise

**DOI:** 10.1186/s12970-021-00467-9

**Published:** 2021-11-07

**Authors:** Yutaka Matsunaga, Kenya Takahashi, Yumiko Takahashi, Hideo Hatta

**Affiliations:** Department of Sports Sciences, The University of Tokyo, 3–8–1 Komaba, Meguro–ku, Tokyo, 153–8902 Japan

**Keywords:** Glycogen recovery, Skeletal muscle, Liver, Glucose metabolism

## Abstract

**Background:**

When a high-carbohydrate diet is ingested, whether as small frequent snacks or as large meals, there is no difference between the two with respect to post-exercise glycogen storage for a period of 24 h. However, the effect of carbohydrate intake frequency on glycogen recovery a few hours after exercise is not clear. Athletes need to recover glycogen quickly after physical exercise as they sometimes exercise multiple times a day. The aim of this study was to determine the effect of carbohydrate intake at different frequencies on glycogen recovery during the first few hours after exercise.

**Methods:**

After 120 min of fasting, 6-week-old male ICR mice were subjected to treadmill running exercise (20 m/min for 60 min) to decrease the levels of muscle and liver glycogen. Mice were then given glucose as a bolus (1.2 mg/g of body weight [BW], immediately after exercise) or as a pulse (1.2 mg/g of BW, every 15 min × 4 times). Following this, the blood, tissue, and exhaled gas samples were collected.

**Results:**

In the bolus group, blood glucose concentration was significantly lower and plasma insulin concentration was significantly higher than those in the pulse group (*p* < 0.05). The plantaris muscle glycogen concentration in the bolus group was 25.3% higher than that in the pulse group at 60 min after glucose ingestion (*p* < 0.05). Liver glycogen concentration in the pulse group was significantly higher than that in the bolus group at 120 min after glucose ingestion (*p* < 0.05).

**Conclusions:**

The present study showed that ingesting a large amount of glucose immediately after exercise increased insulin secretion and enhanced muscle glycogen recovery, whereas frequent and small amounts of glucose intake was shown to enhance liver glycogen recovery.

## Background

Dietary carbohydrates are converted into glycogen, which is stored in the liver and muscles as a major energy source. Carbohydrate utilization depends on exercise intensity [1, 2], and glycogen level decreases with prolonged exercise [3]. Glycogen depletion has several disadvantages, for example, low muscle glycogen level reduces maximal work time [4]. It also modulates the rate of Ca^2+^ release from the sarcoplasmic reticulum (SR) [5], the impairment of which causes force loss [6]. Besides, glycogen plays a key role in supporting Na^+^- and K^+^-ATPases [7] and is, thus, indispensable for muscle contraction and glycogen recovery after exercise.

For efficient post-exercise glycogen recovery, carbohydrate intake in the right amount and timing is necessary. A carbohydrate intake of 1.2 g/kg/h enhances post-exercise muscle glycogen recovery compared with that after an intake of 0.8 g/kg/h in well-trained male cyclists [8]. However, increasing the carbohydrate intake from 1.5 to 3.0 g/kg of body weight (BW) has no additional effect on glycogen recovery in healthy males [9]. Thus, there is an upper limit to the amount of carbohydrate required for glycogen recovery. In fact, Jentjens and Jeukendrup [10] reported that the maximal rate of glycogen synthesis occurs at a carbohydrate intake of ~ 1.2 g/kg/h. Moreover, Ivy et al. [11] reported that a 2 h delay in post-exercise carbohydrate ingestion reduced the rate of muscle glycogen storage compared with that after immediate carbohydrate ingestion in healthy males. In a rodent study, Goodyear et al. [12] reported that 30 min after exercise, glucose uptake is increased and 2 h after exercise glucose uptake returns to the sedentary baseline. Thus, it is important to consume carbohydrates immediately after exercise for efficient glycogen recovery. Whereas, with immediate carbohydrate consumption after exercise, it is not clear how the frequency of carbohydrate intake thereafter affects glycogen repletion. Burke et al. examined the effects of carbohydrate intake at different frequencies on muscle glycogen storage in well-trained triathletes [13]. They reported no difference in post-exercise glycogen storage over a period of 24 h when a high-carbohydrate diet is ingested whether as small frequent snacks or as large meals. Hence, when muscle glycogen recovery time is long, it is not affected by differences in the carbohydrate intake frequency. However, the effect of nutrient intake frequency on glycogen recovery in the early phase, a few hours after exercise, is not clear. Athletes and physically active people need to recover glycogen quickly because they sometimes exercise multiple times a day. Furthermore, it remains unclear whether carbohydrate ingestion frequency affects liver glycogen recovery, although the liver is the main storage tissue for glycogen and contributes to blood glucose homeostasis. Therefore, the aim of this study was to determine the effect of different frequencies of carbohydrate intake on muscle and liver glycogen recovery in the hours after exercise, which may provide useful insights for athletes and physically active people.

## Methods

### Ethical approval

All experimental protocols were approved by the Animal Experimental Committee of The University of Tokyo (No. 28–6).

### Animals

Six-week-old male ICR mice were obtained from CLEA Japan Inc. (Tokyo, Japan). All mice were housed in an environment maintained at 23 °C with a 12/12 h light–dark cycle (dark: 7:00–19:00) and were provided with water and standard chow (3.59 kcal/g; 55.3% carbohydrates, 23.1% protein, 5.1% fat, 5.8% ash, 2.8% fiber, and 7.9% moisture, MF diet) (Oriental Yeast, Tokyo, Japan) ad libitum. They were acclimated for 1 week and were familiarized with the treadmill running exercise at a speed of 15–20 m/min for 10 min for 2 days before starting the experiment.

### Materials

D-glucose and U-^13^C_6_ glucose were obtained (Fujifilm Wako Chemical Corporation) (D-glucose; 047–31,161, U-^13^C_6_ glucose; 574–69,731, Osaka, Japan) and diluted with water to a concentration of 6%.

### Experimental protocols

**Fig. 1:**
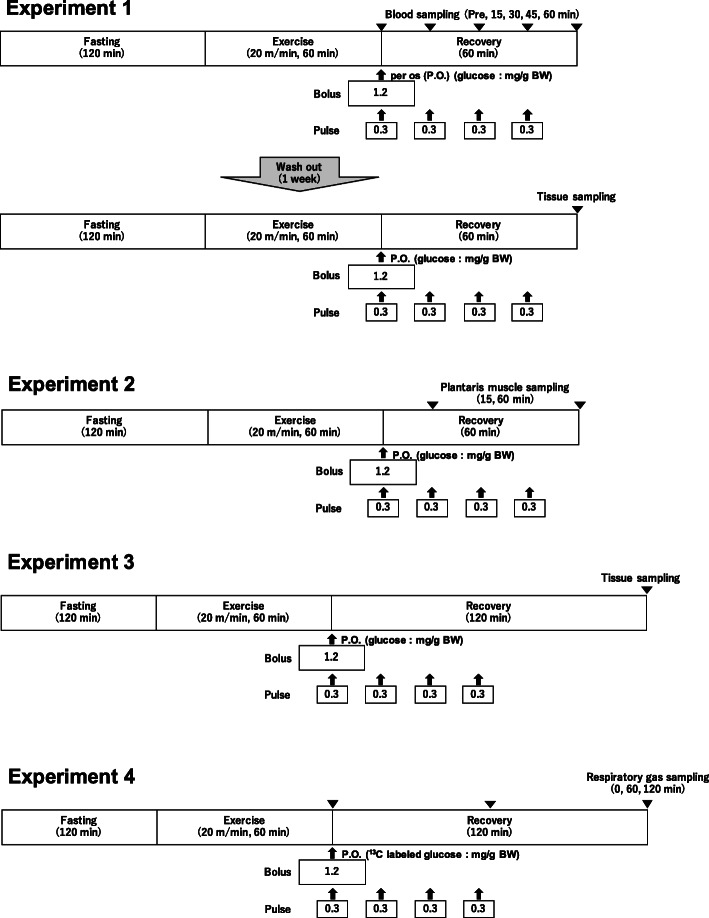
Experimental procedure. Experiment 1: Blood substrate concentrations and tissue glycogen recovery for 60 min after glucose ingestion. Experiment 2: Phosphorylation levels of proteins related to glucose uptake and glycogen synthesis. Experiment 3: Tissue glycogen content at 120 min after glucose ingestion. Experiment 4: Exogenous glucose utilization for 120 min after glucose ingestion

Figure 1 shows a schematic overview of the experimental procedures.

Experiment 1: The effect of different carbohydrate intake frequencies on blood substrate and post-exercise glycogen recovery was investigated. Mice were randomly divided into bolus ingestion group (1.2 mg/g of BW glucose treatment immediately after exercise) and pulse ingestion group (1.2 mg/g BW glucose treatment every 15 min × 4 times). After 120 min of fasting, the mice were subjected to treadmill running exercise (20 m/min for 60 min) to decrease the levels of muscle and liver glycogen. The mice were then administered glucose in bolus or pulse form, and blood samples were collected from the tail vein at 0, 15, 30, 45, and 60 min. Blood samples were then centrifuged (4 °C, 5000×g, 10 min), and the plasma fraction was rapidly frozen in liquid nitrogen and stored at − 80 °C until further analysis. After a 1-week washout period, experiments were performed using a similar protocol. Mice were anaesthetized using isoflurane and euthanized by blood collection at 60 min from the portal vein and inferior vena cava. After collecting 50 μL of blood from the portal vein, the remaining blood samples were collected from the inferior vena cava. The plantaris muscle, soleus muscle, and liver were removed, rapidly frozen in liquid nitrogen, and stored at − 80 °C until further analysis.

Experiment 2: The effects of different ingestion methods on signaling pathways related to glycogen recovery were examined. After performing the same protocol as in experiment 1, the mice were anaesthetized using isoflurane and euthanized by inferior vena cava blood collection. Then, the plantaris muscle and liver were removed, rapidly frozen in liquid nitrogen, and stored at − 80 °C until further analysis. The sampling timings were 15 and 60 min after the first glucose administration in each group.

Experiment 3: The effects of expended recovery time on glycogen content were examined. After 120 min of fasting, mice were subjected to treadmill exercise (20 m/min for 60 min) and glucose was administered in bolus or pulse form. The mice were then allowed to rest for 120 min, were anaesthetized using isoflurane, and euthanized by inferior vena cava blood collection. The plantaris muscle, soleus muscle, and liver were removed, rapidly frozen in liquid nitrogen, and stored at − 80 °C until further analysis.

Experiment 4: To measure exogenous glucose utilization during the recovery period, an exhaled gas analysis using ^13^C glucose was performed. After 120 min of fasting, the mice were subjected to treadmill exercise (20 m/min for 60 min) and administered U-^13^C_6_ stable isotope-labelled glucose (1.2 mg/g BW) in bolus or pulse form. The mice were then placed in a sealed metabolic chamber (MK-680AT, Muromachi Kikai Co. Ltd., Japan). Their exhaled gas was collected (200 mL/min) in the sampling bag using an air pump (Compact Air Station Suction Cas-1, AS ONE, Japan) at 0, 60, and 120 min. Baseline breath samples were collected before the treadmill exercise. The ^13^CO_2_ concentration in the exhaled gas was measured using an infrared spectrophotometer (POCone, Otsuka Electronics Co., Ltd., Japan), following the methods of a previous study [14]. The measured values are presented as ∆^13^CO_2_ (‰). In addition, ∆^13^CO_2_ incremental area under the curve (iAUC) was calculated by summing the area of the increase from the pre-treatment value.

### Blood and plasma substrate concentrations

Blood glucose collected from the tail vein was measured using an auto analyzer (Glutest Ace, Arkray Inc., Kyoto, Japan). Plasma insulin concentration was measured using a Mouse Insulin Enzyme-Linked Immunosorbent Assay Kit (M1102, Morinaga Institute of Biological Science, Inc., Kanagawa, Japan). Portal plasma glucose concentration was measured using a Glucose CII Test Wako Kit (439–90,901, Fujifilm Wako Chemical Corporation, Osaka, Japan). Blood glucose and plasma insulin iAUC was calculated by summing the area of the increase from the pre-treatment value.

### Liver and muscle glycogen concentrations

Glycogen levels in the liver, plantaris muscle, and soleus muscle were measured using the phenol–sulfuric acid method, as described previously [14]. Tissues were weighed and added to 300 μL of 30% KOH with Na_2_SO_4_ to completely dissolve the tissue. The homogenized solutions were mixed with 360 μL ethanol and placed on ice for 30 min, followed by centrifugation (4 °C, 5000×g, 15 min), and the supernatants were removed. The glycogen-containing precipitate was dissolved in distilled water. Phenol and sulfuric acid were added to the solution, the mixture was allowed to react for 15 min, and the absorbance was measured at 490 nm.

### Western blot analysis

The plantaris muscles were homogenized using radioimmunoprecipitation assay lysis buffer (20–188, Millipore, MA, USA) containing a protease inhibitor (1,183,617,001, Complete Mini EDTA-free, Roche Life Science, Indianapolis, IN, USA) and a phosphatase inhibitor (04906837001, PhosSTOP phosphatase inhibitor cocktail, Roche Life Science). The homogenates were placed on ice for 60 min and centrifuged (4 °C, 1500×g, 20 min). The total protein content of the samples was determined using a BCA Protein Assay Kit (23,227, Pierce, Rockford, IL, USA). The proteins (10 μg of each sample) were separated by sodium dodecyl sulphate–polyacrylamide gel electrophoresis. The proteins were then transferred to polyvinylidene difluoride membranes before being blocked for 60 min with 5% [w/v] bovine serum albumin in Tris-buffered saline with 0.1% [v/v] Tween 20 (TBST). Membranes were incubated overnight at 4 °C with the following primary antibodies: phospho-protein kinase B (Akt) (p-Akt Thr308, 9275, Cell Signaling Technology [CST], Tokyo, Japan); phospho-Akt (p-Akt Ser473, 9271, CST); total-Akt (t-Akt, 9272, CST); p-Akt substrate of 160 kDa (AS160) (p-AS160, Thr642, 8881, CST); total-AS160 (t-AS160, 2670, CST); phospho-AMP-activated protein kinase (AMPK) (p-AMPK Thr172, 2535, CST); total-AMPK (t-AMPK, 5832, CST); phospho-glycogen synthase (GS) (p-GS, Ser641, 3891, CST); total-GS (t-GS, 3893, CST); glucose transporter 4 (GLUT4, 07–1404, Merck, Tokyo, Japan); and glyceraldehyde 3-phosphate dehydrogenase (GAPDH, 14C10, 2118, CST). After incubation, the membranes were washed in TBST, incubated for 1 h at room temperature with secondary antibodies (A102PT, American Qualex, CA, USA), and washed again in TBST. Chemiluminescent reagents (RPN 2232 and RPN 2109, GE Healthcare Japan, Tokyo, Japan) were used for blot detection. The blots were scanned and quantified using a ChemiDoc XRS (170–8071, Bio-Rad, Hercules, CA, USA) and Quantity One software (170–9600, Bio-Rad, Hercules, CA, USA).

### Statistical analysis

All data are expressed as the mean ± standard error of the mean. Student’s unpaired t-test and two-way analysis of variance (time × treatment) were performed. The Tukey–Kramer multiple comparisons test was used for post-hoc analysis. The relationship between liver glycogen content and blood substrate concentrations at 120 min after glucose ingestion was assessed by calculating the Pearson’s correlation coefficient. Significant differences were defined as *p*-values < 0.05.

## Results

### Experiment 1

#### Blood glucose and plasma insulin concentrations

**Fig. 2:**
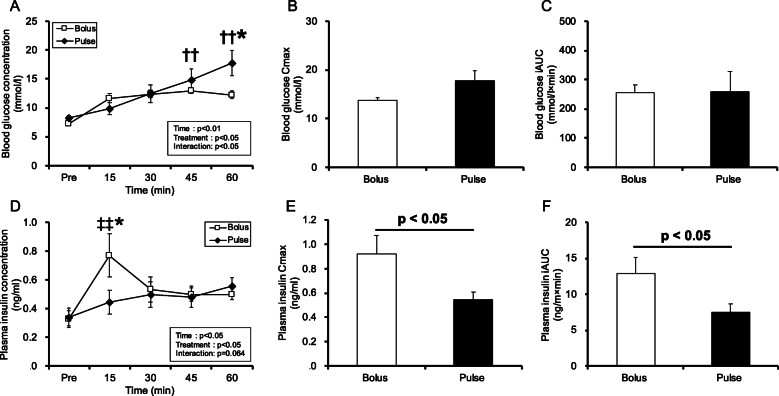
Blood and plasma substrate concentrations. (**A**) Blood glucose concentration; (**B**) Blood glucose Cmax; (**C**) Blood glucose iAUC; (**D**) Plasma insulin concentration; (**E**) Plasma insulin Cmax; (**F**) Plasma insulin iAUC. Values are presented as means ± SEM. * significant difference between the bolus and pulse groups at the same time point (*p* < 0.05). †† significant difference from pre-ingestion in the pulse group (*p* < 0.01). ‡‡ significant difference from pre-ingestion in the bolus group (*p* < 0.05)

Figure 2 shows the time course of the blood substrate concentrations during 60 min of post glucose ingestion. Main effects of time and treatment (time: *p* < 0.01, treatment: *p* < 0.05, Fig. 2A) were observed, and an interaction between time and treatment (*p* < 0.05, Fig. 2A) was observed for blood glucose concentration. Blood glucose concentration in the pulse group at 45 and 60 min was significantly higher than that at pre-ingestion (*p* < 0.01, Fig. 2A). Blood glucose concentration in the bolus group at 60 min was significantly lower than that in the pulse group at 60 min (*p* < 0.05, Fig. 2A). There were no differences in the blood glucose maximum concentration (Cmax) or blood glucose iAUC between the two treatment groups (Fig. 2B-C). There were main effects of time and treatment on plasma insulin concentration (*p* < 0.05, Fig. 2C). Plasma insulin concentration increased in the bolus group at 15 min compared to that at pre-ingestion (*p* < 0.01) and that in the pulse group at 15 min (*p* < 0.05) (Fig. 2D). Plasma insulin Cmax and iAUC in the bolus group were significantly higher than those in the pulse group (*p* < 0.05, Fig. 2E, F).

#### Glycogen concentrations

**Fig. 3:**
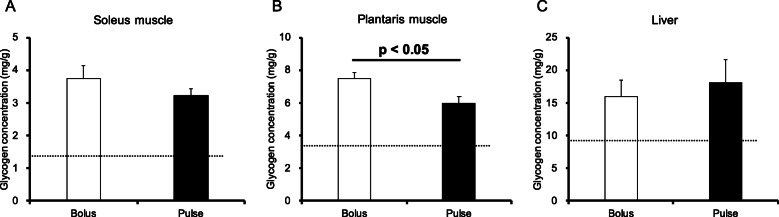
Glycogen content at 60 min after glucose ingestion in muscle and liver. (**A**) Soleus muscle glycogen concentration; (**B**) Plantaris muscle glycogen concentration; (**C**) Liver glycogen concentration. Values are presented as means ± SEM

Glycogen concentrations in the sedentary group were as follows: soleus, 5.3 ± 0.4; plantaris, 6.0 ± 0.4; and liver, 49.4 ± 1.8 mg/g wet weight mass. Glycogen concentrations in the post-exercise group were as follows: soleus, 1.4 ± 0.1; plantaris, 3.4 ± 0.2; and liver, 9.2 ± 3.7 mg/g wet weight mass. These results indicated that endurance exercise in this study reduced muscle and liver glycogen concentrations. At 60 min after the exercise, the soleus muscle glycogen content in the bolus group was 16.2% higher than that in the pulse group, but the difference was not statistically significant (Fig. 3A). The plantaris muscle glycogen concentration in the bolus group was 25.3% higher than that in the pulse group (*p* < 0.05, Fig. 3B). There were no significant differences in liver glycogen concentrations between the two groups (Fig. 3C).

#### Portal plasma glucose concentrations

**Fig. 4:**
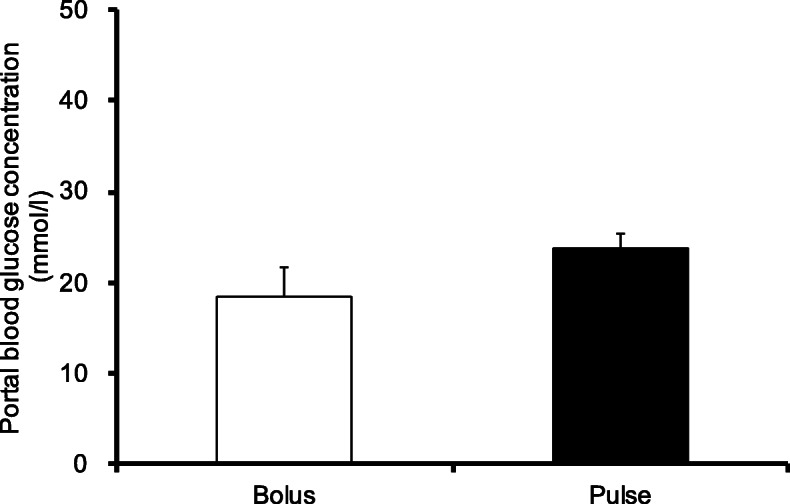
Portal plasma glucose concentration. Values are presented as means ± SEM

Given that glucose is absorbed in the small intestine and transported to the liver through the portal vein, we next evaluated portal plasma glucose concentrations. The portal plasma glucose concentration at 60 min after the exercise did not differ between the two groups (Fig. 4).

### Experiment 2

#### Signaling pathways

**Fig. 5:**
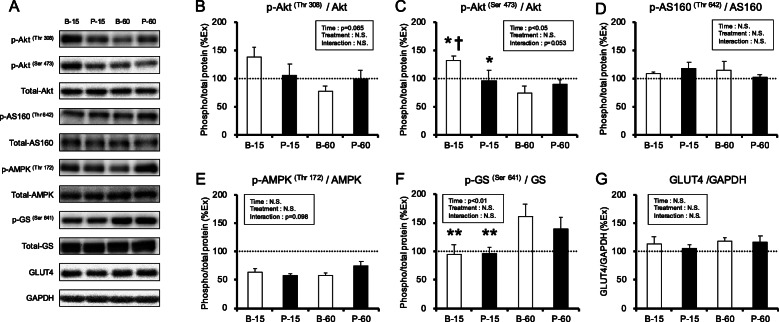
Phosphorylated protein levels in plantaris muscle at 15 and 60 min after glucose ingestion. (**A**) Representative western blots for p-Akt^Thr308^, p-Akt^Ser473^, t-Akt, p-AS160^Thr642^, t-AS160, p-AMPK^Thr172^, t-AMPK, p-GS^Ser641^, t-GS, GLUT4, and GAPDH; (**B**) p-Akt^Thr308^/t-Akt ratio; (**C**) p-Akt^Ser473^ /t-Akt ratio; (D) p-AS160^Thr642^ /t-AS160 ratio; (**E**) p-AMPK^Thr172^ /t-AMPK ratio; (**F**) p-GS^Ser641^ /t-GS ratio; (**G**) GLUT4 protein level. Values are presented as means ± SEM. Post-exercise group was presented as a dotted line. * main effect of time (*p* < 0.05). ** main effect of time (*p* < 0.01). †significant difference from the bolus at 60 min (B-60) group (*p* < 0.05)

To clarify the mechanism of different administration methods on glycogen recovery, signaling pathways in the plantaris muscle were examined. Figure 5 shows representative western blots for Akt ^(Thr308)^, Akt ^(Ser473)^, AS160 ^(Thr642)^, AMPK ^(Thr172)^, GS ^(Ser641)^, and GLUT4 (Fig. 5A). The phosphorylation state of Akt ^(Thr 308)^ was not significantly different among the groups (Fig. 5B). Time had a significant effect on Akt ^(Ser473)^ phosphorylation (*p* < 0.05). The phosphorylation state of Akt ^(Ser473)^ in the bolus group at 15 min (B-15) was significantly higher than that in the group at 60 min (B-60) (*p* < 0.05, Fig. 5C), while that of AS160 ^(Thr642)^ was similar among these groups (Fig. 5D). No differences in AMPK ^(Thr172)^ phosphorylation were observed among the groups (Fig. 5E). Time had a significant effect on GS ^(Ser641)^ phosphorylation (*p* < 0.01, Fig. 5F). GLUT4 protein content was similar among the groups (Fig. 5G).

### Experiment 3

#### Glycogen recovery 120 min after glucose ingestion

**Fig. 6:**
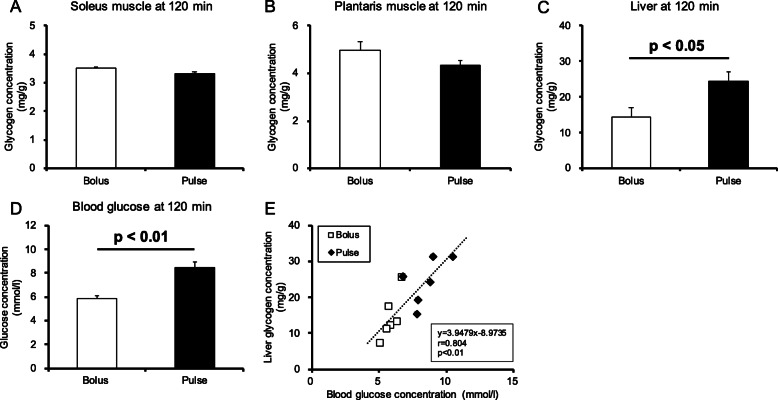
Blood glucose and tissue glycogen concentrations at 120 min after glucose ingestion. (**A**) Soleus muscle glycogen concentration; (**B**) Plantaris muscle glycogen concentration; (**C**) Liver glycogen concentration; (**D**) Blood glucose concentration; (**E**) Correlation between blood glucose concentration and liver glycogen concentration. Values are presented as means ± SEM

The bolus group was administered glucose immediately after exercise, whereas the pulse group was administered glucose in four separate doses. The possibility of delay in glycogen recovery in the pulse group could not be dismissed due to insufficient absorption of the administered glucose. Therefore, glycogen levels were confirmed when the recovery time was extended to 120 min. The soleus and plantaris muscle glycogen contents did not differ among the groups (Fig. 6A-B). In contrast to skeletal muscle, liver glycogen content in the pulse group was significantly higher than that in the bolus group (Fig. 6C, *p* < 0.05). Similarly, blood glucose concentration in the pulse group at 120 min was higher than that in the bolus group (Fig. 6D, *p* < 0.01). In addition, a significant positive correlation was observed between blood glucose concentration and liver glycogen content at 120 min after glucose ingestion (Fig. 6E, *r* = 0.804, *p* < 0.01).

### Experiment 4

#### Exogenous glucose utilization during glycogen recovery period

**Fig. 7:**
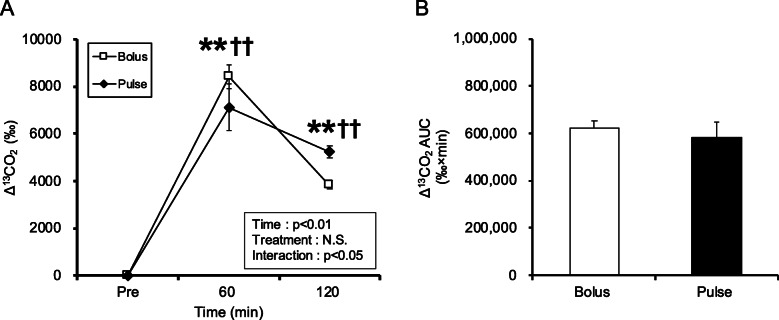
Exogenous glucose utilization for 120 min after glucose ingestion. (**A**) Amount of change in ^13^CO_2_; (**B**) Δ^13^CO_2_ iAUC. Values are presented as means ± SEM. ** significant difference from pre-ingestion in the pulse group (*p* < 0.01). ††significant difference from pre-ingestion in the bolus group (*p* < 0.01)

In this study, we examined glycogen synthesis and also measured the utilization of exogenous glucose. A main effect of time and an interaction between time and treatment was observed for Δ^13^CO_2_ (time: *p* < 0.01, interaction: *p* < 0.05, Fig. 7A). Although Δ^13^CO_2_ in both groups was significantly higher than that in the pre-ingestion period (*p* < 0.01, Fig. 7A), there were no differences among the groups. The Δ^13^CO_2_ iAUC was similar between the two groups (Fig. 7B).

## Discussion

To the best of our knowledge, this is the first study to determine the effect of differences in the frequency of carbohydrate intake on glycogen recovery in the early phase post-exercise. Our results showed that bolus (high dose and single administration immediately after the exercise) glucose intake enhanced post-exercise muscle glycogen repletion in the plantaris muscle as compared to pulsed (low dose and frequent administration during the post-exercise phase) glucose intake. In contrast, liver glycogen repletion was enhanced by pulsed glucose intake compared with that after bolus glucose intake.

Glycogen is mainly stored in the liver and skeletal muscles. Glucose transport into skeletal muscles is mediated by the GLUT1 and GLUT4 proteins [15]. GLUT1 is found in the plasma membrane and contributes to basal glucose transport [16], while the more abundant GLUT4 is largely sequestered intracellularly from where it is rapidly translocated to the cell surface in response to insulin and exercise [16]. Insulin-stimulated glucose uptake into the muscles is enhanced by exercise [17]. Increased insulin secretion enhances skeletal muscle glycogen recovery after exercise [18]. Hence, it is important to increase insulin secretion after exercise. In this investigation, plasma insulin concentration in the bolus group was significantly higher than that in the pulse group (Fig. 2). Blood glucose concentration in the bolus group was, thus, significantly lower than that in the pulse group at 60 and 120 min (Figs. 2 and 6). Our results suggest that a high amount of glucose ingestion is required at once to increase insulin secretion and promote glucose uptake. Furthermore, Akt phosphorylation was higher in the B-15 group than in the B-60 group. However, there was no difference among the B-60, P-15, and P-60 groups in this study (Fig. 5). Akt pathway plays an important role in glucose uptake into the muscles [19]. Therefore, it is possible that activation of Akt phosphorylation by increased insulin secretion may be partly responsible for higher muscle glycogen recovery in the bolus group than in the pulse group.

A previous study reported that activating gluconeogenesis and inactivating glycolysis enhanced glycogen repletion in the skeletal muscle and liver [20]. Furthermore, we previously reported that enhancement of energy expenditure during the post-exercise period suppressed glycogen recovery [21]. Therefore, it is necessary to consider glycogen recovery in terms of not only glucose uptake and glycogen synthesis but also glucose consumption. In this experiment, ^13^C glucose was used to assess the utilization of exogenous glucose, but no difference was found between the two groups i.e., the bolus and pulse groups (Fig. 7). Thus, differences in mode of glucose administration may not affect the utilization of exogenous glucose during the glycogen recovery period. Furthermore, increased meal frequency might slow small-intestinal absorption of glucose [22]. However, portal glucose concentration at 60 min after glucose ingestion did not differ between the two groups (Fig. 4). The portal vein is the blood vessel that supplies nutrients absorbed in the small intestine to the liver and is thought to reflect the state of absorbed nutrients. In addition, even when the recovery time was extended to 120 min, the muscle glycogen concentration in pulse group did not exceed that of the bolus group. Consequently, it is likely that other factors and not the time of absorption caused the difference in muscle glycogen recovery.

Liver glycogen is essential for the maintenance of blood glucose levels [23]. Hepatic glycogenolysis accounts for approximately 45% and gluconeogenesis accounts for approximately 55% of the whole-body glucose production [24]. Hepatic oxygen consumption increases during exercise, resulting in an enhanced liver metabolic rate and glucose utilization [25]. Liver glycogen is depleted by prolonged exercise, whereby liver glucose output and muscle glucose uptake are reduced [26]. It is thought that enhancing glycogen recovery in the liver is important to ensure a stable energy supply into the skeletal muscle. Increased postprandial insulin release contributes to glucose uptake and glycogen recovery in the liver [25]. Thus, we expected liver glycogen to be higher in the bolus group than in the pulse group, similar to the trend observed in the skeletal muscles. However, interestingly, liver glycogen concentration at 120 min was higher in the pulse group than in the bolus group. In the bolus group, plasma insulin concentrations at 15 min were higher but returned to the same levels as those in the pulse group at 30 min and afterwards. Muscle glycogen recovery occurs preferentially to liver glycogen after exercise [27]. It is assumed that increase in plasma insulin concentration during early post-exercise phase facilitates glycogen repletion in the muscles more than in the liver. In addition, the liver expresses GLUT2, which is responsible for glucose transport [28]. As GLUT2 has a low glucose affinity, an elevation in postprandial blood glucose concentration enhances the rate of glucose transport and intracellular glucose concentration [29]. Given the results of this study, the pulse group possibly had less glucose uptake by the skeletal muscle, resulting in increased glucose influx to the liver and, thus, higher liver glycogen recovery than the bolus group.

In this study, we used mice because it is difficult to obtain muscle and liver samples from human subjects. Thus, our findings may not be directly applied to humans. However, previous studies reported that glycogen levels decreased with prolonged exercise [3, 21, 30] and were then restored with nutrient intake [18, 31] in both human and rodent studies. In addition, muscle glycogen reduction impaired endurance performance in human and animal subjects [4, 32] and enhancing liver glycogen concentration increased exercise capacity in mice [33]. Furthermore, enhancement of glycogen resynthesis after exercise affects subsequent exercise performance in healthy males [34], healthy recreationally active people [35], and endurance-trained male cyclists [36]. For instance, Alghannam et al. [35] reported that high carbohydrate drink intake enhanced glycogen repletion by 73% and subsequent endurance exercise duration by 66% compared to low carbohydrate drink intake. Given that glycogen is an essential energy substrate in both humans and rodents, the results of this study may be useful for athlete and physically active people. Our observations showed that bolus glucose ingestion enhanced muscle glycogen recovery compared to the pulse ingestion. A previous study reported that muscle glycogen utilization is higher in exercise with intensity above 65% VO2 max [1]. Thus, bolus glucose intake is recommended for practitioners who perform high-intensity exercise. In this study, pulse glucose intake increased liver glycogen recovery. Therefore, it may be suitable for practitioners who perform low-intensity prolonged exercise. In addition, a previous study reported that glucose absorption within the intestinal segment was estimated to range from 1.3 to 1.7 g glucose/min in sedentary healthy males [37], whereas gastric emptying rate was reported to be decreased after a single bout of strenuous resistance exercises in healthy young subjects [38]. Therefore, people who tend to have digestive problems after exercise or who have digestive disorders may benefit by pulse glucose ingestion.

## Conclusions

The present study examined the effects of different methods of post-exercise glucose intake on early glycogen recovery. Single ingestion of a large amount of glucose immediately after exercise increased insulin secretion and enhanced muscle glycogen recovery. In contrast, frequent and small amounts of glucose intake was shown to enhance glycogen recovery in the liver. However, there was no difference in glucose utilization. The results of this study are expected to add to the literature regarding glucose uptake and glycogen synthesis, but the detailed mechanism requires further investigation.

## Data Availability

All data generated or analyzed during this study are included in this published article.

## Abbreviations

Akt: Protein kinase B
AMPK: AMP-activated protein kinase
AS160: Akt substrate of 160 kDa
BW: Body weight
Cmax: maximum concentration
GAPDH: Glyceraldehyde 3-phosphate dehydrogenase
GLUT: Glucose transporter
GS: Glycogen synthase
iAUC: Incremental area under the curve
